# Reciprocal cortical activation patterns during incisal and molar biting correlated with bite force levels: an fMRI study

**DOI:** 10.1038/s41598-019-44846-4

**Published:** 2019-06-10

**Authors:** Hideyuki Yoshizawa, Jun J. Miyamoto, Takashi Hanakawa, Hitoshi Shitara, Manabu Honda, Keiji Moriyama

**Affiliations:** 1 Department of Maxillofacial Orthognathics, Division of Maxillofacial and Neck Reconstruction, Graduate School of Medical and Dental Sciences, Tokyo Medical and Dental University (TMDU), Tokyo, Japan; 2 Department of Functional Brain Research, National Institute of Neuroscience, National Center of Neurology and Psychiatry, Tokyo, Japan; 3 Department of Advanced Neuroimaging, Integrative Brain Imaging Center, National Center of Neurology and Psychiatry, Tokyo, Japan; 4 Department of Orthopaedic Surgery, Gunma University Graduate School of Medicine, Maebashi, Japan

**Keywords:** Motor control, Oral anatomy

## Abstract

In humans, the incisors and molars have distinct functions during mastication, analogous to the two main types of handgrip, the precision and power grips. In the present study, we investigated cortical activation and masticatory muscle activity during incisal and molar biting via simultaneous functional magnetic resonance imaging and electromyogram (EMG) recordings. We conducted recordings in 15 healthy adult participants while they performed incisal and molar biting tasks at three step-wise force levels using two custom-made splints. Regarding the results of the ROI analysis, we found a significantly stronger positive linear correlation between the blood oxygenation level dependent signal and EMG activity during molar biting than incisal biting, which was particularly prominent in the primary sensorimotor cortex and the cerebellum. We also found a significantly stronger negative linear correlation during incisal biting than molar biting, which was particularly prominent in the rostral cingulate motor area, superior frontal gyrus, and caudate nucleus. These findings indicate that molar biting enables powerful chewing: brain activity in several brain areas related to motor function was increased with increasing bite force levels, while incisal biting enables fine motor control: brain activity in several brain areas related to motor control was increased with reduced bite force levels.

## Introduction

Among tetrapods, the forelimbs and mouth are functionally similar in that they are both used to perform ‘grasping’ behaviours^1^. In mammals, mothers grasp their infants via two main behaviours (infant carrying): fur-grasping and oral carrying. These are performed by the forelimbs and mouth, respectively^2,3^. Mammals also obtain and transport food or prey via grasping and reaching behaviours with their hands and mouths (e.g. microcebus murinus)^4^. A previous neurophysiological study discovered a class of neurons in the monkey premotor cortex (area F5) that fired when the animal grasped an object with both its hands and mouth^5^. The hands and mouth are considered to engage in a sequence of corresponding movements that enable an individual to achieve the final goal of ingesting food^6^. Furthermore, a behavioural study in humans indicated that grasping movements made with the hands or the mouth can affect movement in other distal effectors^7^. Thus, the hands and mouth may play similar roles in ‘grasping behaviours’ in humans.

The morphology of handgrips during grasping behaviour has taken various forms over the course of primate evolution^8,9^. In humans, there are two fundamental categories of handgrip: the power grip and the precision grip. During a power grip, all of the fingers on a hand participate in grasping an object against the palm using a large force. In a precision grip, contact with the object is confined to the pulps of the index finger and thumb, involving a smaller amount of force^10^. The power grip is typically involved in powerful grasping. Previous functional magnetic resonance imaging (fMRI) studies using power-grip tasks have reported an association between power gripping and brain activation in the primary sensorimotor cortex (M1S1) and cerebellum^11,12^. The precision grip plays a key role in fine motor control, and previous fMRI studies using dexterous precision-grip tasks have reported an association between precision gripping and brain activation in the rostral cingulate motor area (CMAr)^13^, superior frontal gyrus^13,14^, and basal ganglia^15^. Moreover, several previous fMRI studies using the power-grip task reported that brain activation in the M1S1 and cerebellum were stronger during a high grip force condition compared with a low grip force condition^16–18^, while another previous fMRI study using the precision-grip task reported that brain activation in the CMAr was stronger in a low grip force condition compared with a high grip force condition^19^. Thus, the generation of grip forces for these two handgrip types appears to be associated with different patterns of brain activity.

In addition to handgrip behaviour, vertebrates exhibit remarkable diversity in tooth shape, which reflects evolutionary adaptation to new foods, and thus new feeding strategies^20^. While reptiles have homodont dentition involving conical teeth with a simple structure, mammals developed heterodont dentition in the course of evolution^21^. In humans, there are two main tooth shapes: molar and incisor. Generally, molars are thought to have evolved from conical teeth via the progressive addition of extra cones, whereas incisors are unique tooth type neither conical nor multicuspid^20,22^. The two teeth types also differ in terms of function: molars play a role in crushing and grinding food, while incisors are involved in procuring and ingesting food^22^. In addition, dynamic sensitivity for periodontal afferents is lower in posterior vs. anterior teeth^23^, such that incisors can play a key role in fine motor control. Therefore, considering the above-mentioned analogy between the hands and mouth in ‘grasping’ behaviours, biting patterns may differ between molars and incisors in terms of motor control, such as power and precision gripping ability. However, the motor control systems operating during incisal and molar biting are not clearly understood.

In everyday life, incisal and molar biting play specific roles in masticatory function. Masticatory function has been evaluated using various methods, including chewing cycle, occlusal contact, and bite force^24,25^. Although some of these methods can evaluate the function of molar biting during actual operation, each test can evaluate only one peripheral capability of mastication (e.g., the measurement of particle size after crushing and grinding food^26^, and the measurement of changes in the colour tone of gum after mixing two different colours by chewing^27^). In addition, there is currently no method for evaluating the function of incisal biting (ingesting and cutting food) including fine motor control while actually operating. Thus, observing the cortical motor control systems may newly enable evaluation of the comprehensive functions of each type of tooth during actual operation. To the best of our knowledge, no previous studies have examined the motor control systems engaged during incisal and molar biting from the viewpoint of brain function.

The purpose of the present study was to elucidate the difference between motor control systems during incisal and molar biting. Specifically, we examined whether the brain activation patterns corresponding to force levels differed between two types of biting, similar to the differences between precision and power gripping. We hypothesised that brain activation in the M1S1 and cerebellum would increase with increasing bite force levels during molar biting, similar to power grip. In contrast, we hypothesised that activation in the CMAr would increase with reducing bite force levels, and that brain activity in the CMAr, superior frontal gyrus, and basal ganglia, which are reported to be associated with fine motor control^13–15^, would be found in the lower force condition during incisal biting, similar to precision grip. To achieve this purpose, we investigated the masticatory muscle activity and cortical activation that occurs during incisal and molar biting by recording electromyogram (EMG) and fMRI signals simultaneously in humans. Then, we examined the ways in which cortical activation covaried with EMG activity to clarify how brain activation varied according to three step-wise bite force levels (soft, medium, and hard).

## Results

### EMG data

**Figure 1:**
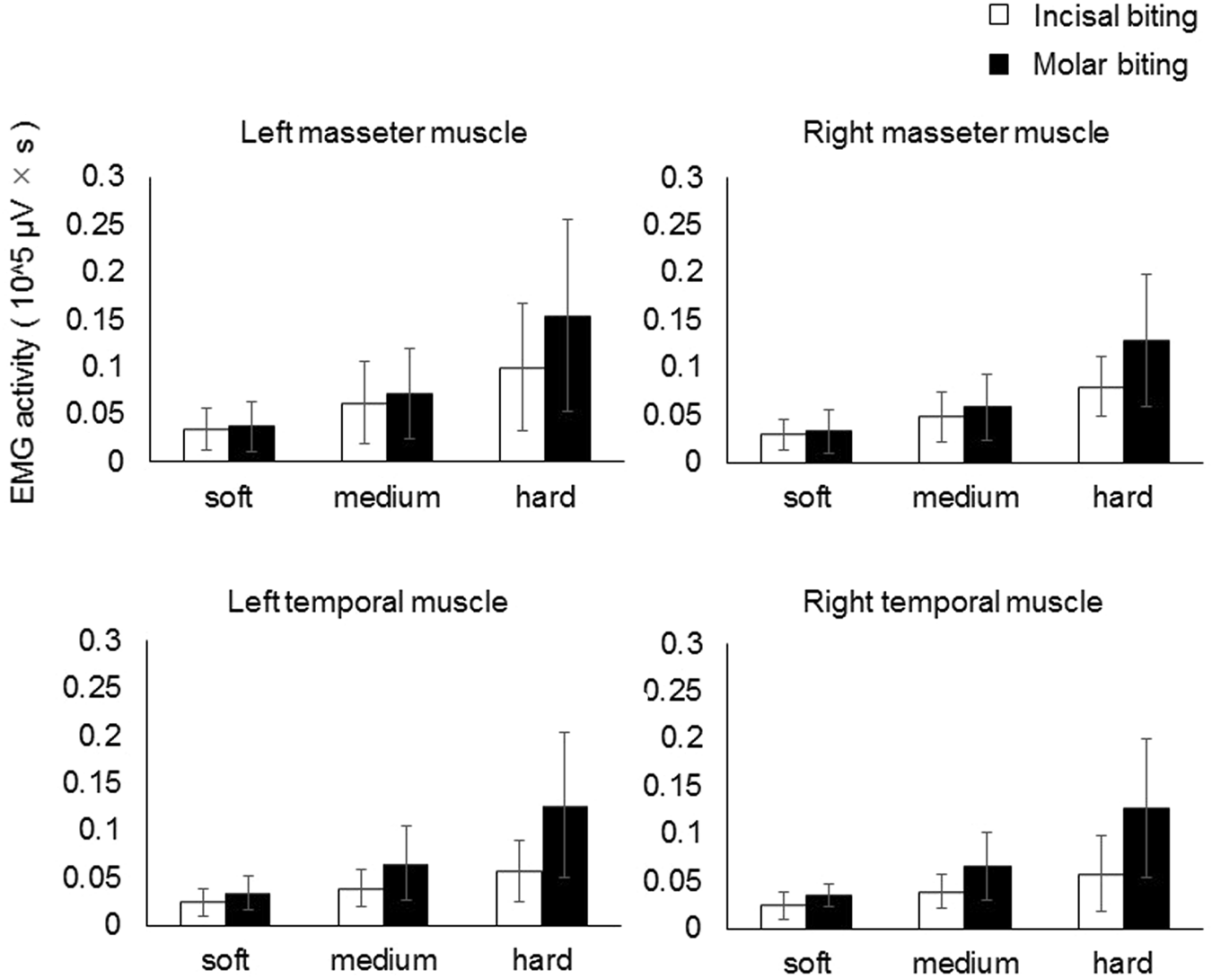
EMG activity for each bite type and intensity condition (means and SDs). Two-way repeated ANOVA showed significant differences between EMG activity in the three bite force conditions (soft, medium, hard) for all four muscles (significant at P < 0.05). A *post hoc* analysis using a paired t-test accompanied by a Bonferroni-Holm correction revealed significant differences between the soft and medium conditions, between the soft and hard conditions, and between the medium and hard conditions in all four muscles (significant at adjusted P < 0.05). ANOVA also revealed significant differences in EMG activity between incisal and molar biting in the right masseter muscle, left temporal muscle, and right temporal muscle (significant at P < 0.05).

Figure 1 shows the mean value and standard deviation of the integral of EMG activity per one bite in the bilateral masseter and temporal muscles in each condition. We found significant differences between EMG activity in the three bite force conditions (soft, medium, and hard) for the left masseter muscle [F (5, 84) = 19.236, P < 0.001], right masseter muscle [F (5, 84) = 29.768, P < 0.001], left temporal muscle [F (5, 84) = 19.436, P < 0.001], and right temporal muscle [F (5, 84) = 19.550, P < 0.001]. A *post hoc* analysis using a paired t-test accompanied by a Bonferroni-Holm correction revealed significant differences between the soft and medium conditions, between the soft and hard conditions, and between the medium and hard conditions in all four muscles. There were significant differences in EMG activity in the right masseter muscle [F (5, 84) = 6.725, P = 0.011], left temporal muscle [F (5, 84) = 18.336, P < 0.001], and right temporal muscle [F (5, 84) = 18.336, P < 0.001] between incisal and molar biting.

These main effects were qualified by significant interactions in the left [F (5, 84) = 4.451, P = 0.015] and right temporal muscles [F (5, 84) = 4.658, P = 0.012], so we investigated the simple main effects. As for the biting position, a simple main effects comparison found significant differences between EMG activities in the incisal and molar biting trials in the hard condition, specifically in the left [F (1, 84) = 22.263, P < 0.001] and right temporal muscles [F (1, 84) = 24.563, P < 0.001], whereas no significant differences were found in the soft and medium conditions.

### Region of interest (ROI) analysis data

**Figure 2:**
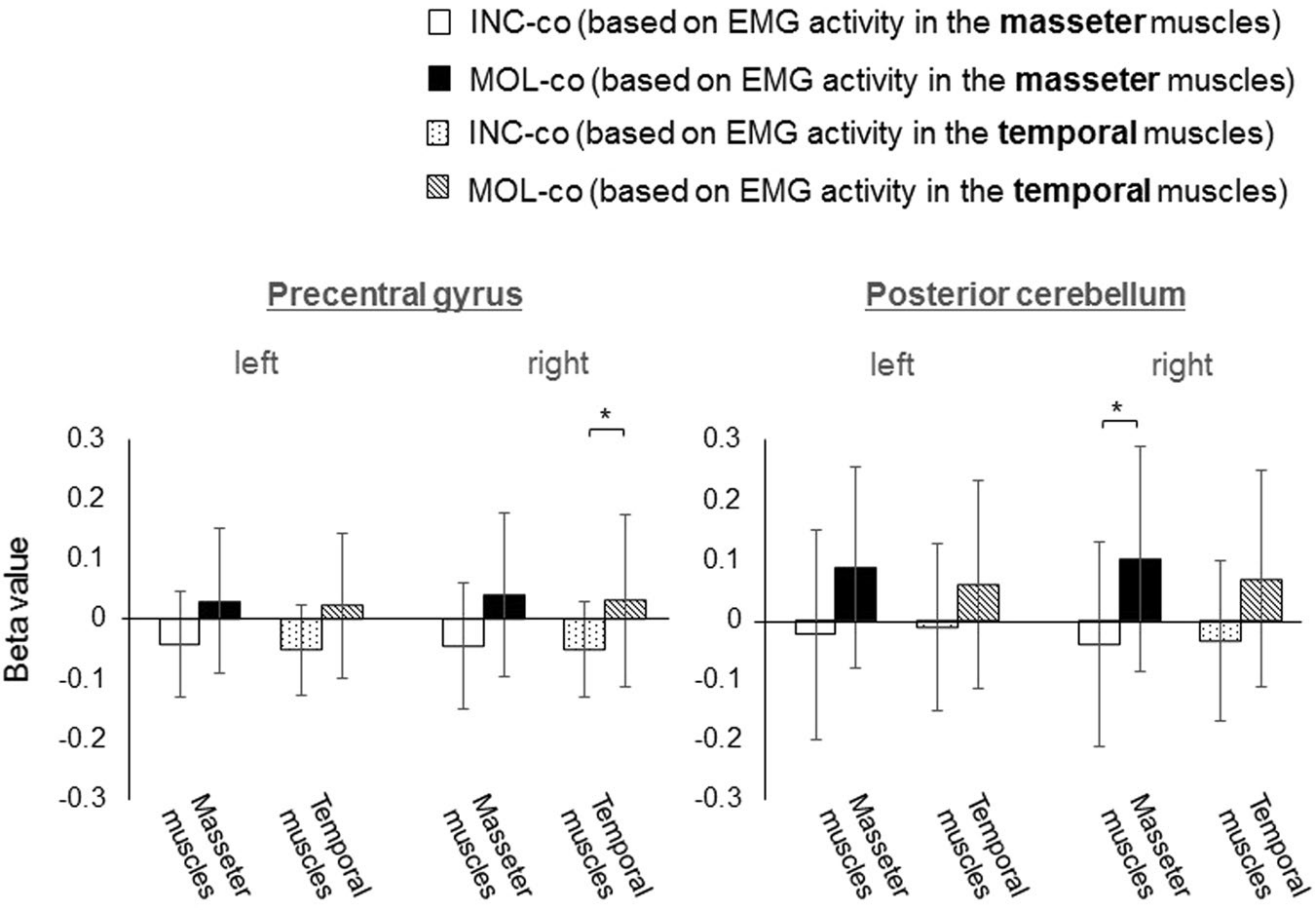
Beta values for the contrast of INC-co and MOL-co in each anatomical ROI for bilateral precentral gyrus and posterior cerebellum. (Means and SDs); *P < 0.05. The results of the ROI analysis in left hemisphere (the left graph), and right hemisphere (the right graph) were shown for each ROI. The contrasts of INC-co and MOL-co were based on EMG activity of the masseter muscles and temporal muscles.

**Figure 3:**
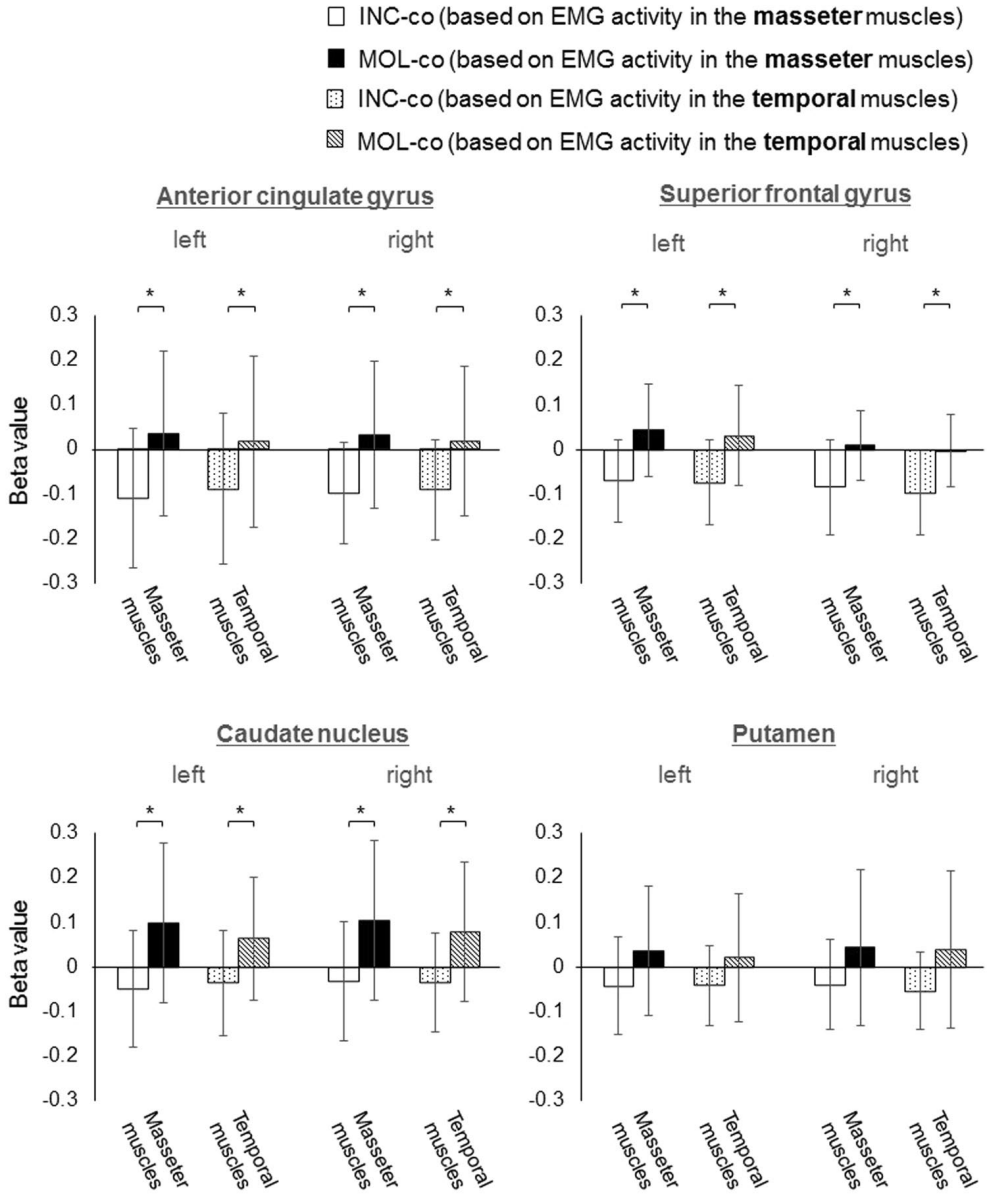
Beta values for the contrast of INC-co and MOL-co in each anatomical ROI for bilateral anterior cingulate gyrus, superior frontal gyrus, caudate nucleus, and putamen. (Means and SDs); *P < 0.05. The results of the ROI analysis in left hemisphere (the left graph), and right hemisphere (the right graph) were shown for each ROI. The contrasts of INC-co and MOL-co were based on EMG activity of the masseter muscles and temporal muscles.

Differences between brain activation covaried with EMG activity during incisal biting (INC-co) and that during molar biting (MOL-co) were examined using ROI analysis of beta values, each of which was obtained from the contrast of INC-co or MOL-co in the general linear model using statistical parametric mapping software (SPM12; http://www.fl.ion.ucl.ac.uk/spm). Figure 2 shows the beta values of the ROI analysis for the bilateral precentral gyrus and posterior cerebellum. Regarding activation that was correlated with EMG activity in the masseter muscles, the beta values in MOL-co were significantly higher than those in INC-co in right posterior cerebellum (T = 2.321, P = 0.036). Regarding activation that was correlated with EMG activity in the temporal muscles, the beta values in MOL-co were significantly higher than those in INC-co in the right precentral gyrus (T = 2.362, P = 0.033) (Fig. 2). Figure 3 shows the beta values for the bilateral anterior cingulate gyrus (including the CMAr), superior frontal gyrus, (including BA 6 and 9), caudate nucleus, and putamen. Regarding activation that was correlated with EMG activity both in the masseter muscles and the temporal muscles, the beta values in INC-co were significantly lower than the MOL-co values in the bilateral anterior cingulate gyrus (right; [masseter muscle; T = 3.208, P = 0.006, temporal muscle; T = 2.853, P = 0.013)], left; [masseter muscle; T = 2.804, P = 0.014, temporal muscle; T = 2.478, P = 0.027]), bilateral superior frontal gyrus (right; [masseter muscle; T = 2.812, P = 0.014, temporal muscle; T = 3.174, P = 0.007], left; [masseter muscle; T = 3.479. P = 0.002, temporal muscle; T = 3.007, P = 0.014), and bilateral caudate nucleus (right; [masseter muscle; T = 2.635, P = 0.002, temporal muscle; T = 2.805, P = 0.014], left; [masseter muscle; T = 2.789, P = 0.014, temporal muscle; T = 2.175, P = 0.047]) (Fig. 3).

### Voxel-wise fMRI data (for illustration purposes)

**Figure 4:**
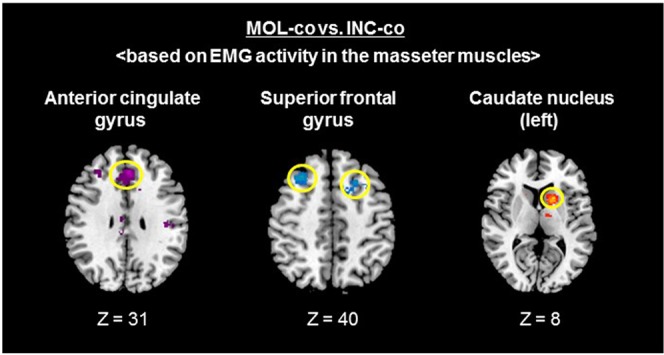
Activated brain areas for the contrast of MOL-co vs. INC-co. For the purposes of illustration, all activations are shown at P < 0.005 (uncorrected). Transverse brain slices of group-activated brain areas for the contrast of MOL-co vs. INC-co. The contrasts of MOL-co and INC-co were based on EMG activity in the masseter muscles.

We examined interactions among the brain activation patterns that covaried with EMG activity between during incisal and molar biting using voxel-wise analysis for illustration purposes. We found activation in the bilateral cingulate gyrus, bilateral superior frontal gyrus, and left caudate nucleus in the contrast of MOL-co vs. INC-co based on EMG activity in the masseter muscles (P < 0.005, uncorrected) (Fig. 4). Each of the selected ROIs was not a singularly isolated point, but formed a cluster with a unique spread, supporting the validity of the ROI selection.

## Discussion

We investigated differences in motor control systems between incisal and molar biting by recording fMRI and EMG data from masseter and temporal muscles. We produced two main findings: (1) during molar biting, the BOLD signal and EMG activity of the masseter and temporal muscles were positively linearly correlated, and (2), during incisal biting, the BOLD signal and EMG activity in the masseter and temporal muscles were negatively linearly correlated.

Regarding EMG results, we found significant differences in EMG activities between the three bite force conditions (soft, medium, and hard) in all four examined muscles. Thus, we considered that the participants had been able to appropriately adapt their bite force levels according to our instructions. Importantly, we did not use visual feedback regarding exerted force during the experimental period because we sought to investigate how bite force levels were controlled by somatosensory information only, which we considered to be more comparable to a natural chewing situation. A previous study revealed that visual feedback was more helpful for maintaining a constant biting force than producing varied force levels, and that periodontal afferent inputs (somatosensory information) were important for controlling force levels, while visual feedback was helpful for both maintaining a constant grip force and producing varied force levels during the pinch grip task^28^. Thus, the current results revealed that participants could vary their bite force levels using only somatosensory information.

In the bilateral temporal muscles, our simple main effects comparison showed significant differences in EMG activities between incisal and molar biting in the hard force condition. This result is consistent with that of a previous study that demonstrated that the difference in occlusal force between incisal and molar biting increased as clenching intensity increased^29^.

As for differences in the changes in EMG activity corresponding to bite force levels between incisal and molar biting, our EMG results showed a significant interaction in EMG activity in the temporal muscles, and no interaction in EMG activity in the masseter muscles. This may indicate that the regulation strategy for masseter muscle activity corresponding to the three bite force levels was similar in incisal and molar biting, while that for temporal muscle activity was different in incisal and molar biting. A previous EMG study found that, during incisal biting, EMG activities in the masseter and temporal muscles were similar at 50% of the maximum voluntary contraction, whereas EMG activity in the masseter muscles was higher than that in the temporal muscles during submaximal incisal biting. This previous result indicates that temporal muscle activities are inhibited and masseter muscle activities are predominant during incisal biting with a high force level^30^. Conversely, during molar biting, the temporal muscle is active even during submaximal clenching^30^. Therefore, the control system for masticatory muscle activities may differ between the incisal and molar biting task.

Regarding the results of brain activation showing a linear positive correlation with EMG activity, the beta value of MOL-co, particularly for the ROI defined in the right M1S1 and right cerebellum, was significantly greater than that for INC-co. Several previous fMRI studies using the power-grip task have shown that BOLD signals in M1S1 and the cerebellum linearly increase as the applied force increases^16–18^. Hence, concerning the relationship between exerted force and cerebral activity, power grip and molar biting tasks appear to have similar tendencies.

As for the M1S1, previous studies have reported that a higher power-grip force increased the firing rate of primary motor cortex neurons in conscious monkeys^31,32^ and caused increased somatosensory feedback because of stronger stimulation of cutaneous receptors and proprioceptors^33^. This may also be the case for molar biting. As the cerebellum is thought to be a place where sensory and motor information converge to produce voluntary movement, there would be a cerebello-cortical loop between the cerebellum and M1S1^34^. These previous data support our observation of a linear increase in activation in the M1S1 and cerebellum with corresponding EMG activity during a molar biting task.

We found that the beta values of INC-co (based on EMG activity in both the masseter and temporal muscles), particularly for the ROIs defined in the bilateral CMAr, superior frontal gyrus, and caudate nucleus were significantly lower than those of MOL-co. A previous study using the precision-grip task showed stronger brain activation in the CMAr in a low grip force vs. high grip force condition^19^. Thus, the precision grip and incisal biting tasks appear to carry similar relationships with respect to exerted force and brain activity. This result may indicate that the linear decrease in brain activation corresponding to the increase in EMG activity is characteristic in incisal biting.

The CMAr was reported to be more strongly activated in a lower precision-grip force condition^19^. As a result, this region was considered to be involved in force control during fine-skilled precision grip actions^11,19^. Furthermore, a previous fMRI study showed that the CMAr was more strongly activated when participants held an object using a reduced but controlled (without slipping) grip force (gentle grip), compared with when they held the object firmly (firm grip). This indicates that the CMAr is associated with fine motor control tasks^13^.

The superior frontal gyrus region (BA6), for which activation was negatively correlated with EMG activity in the temporal muscles, is located in the dorsal premotor area (PMd). In a previous study using grip and lift tasks, transient virtual lesions of the PMd via transcranial magnetic stimulation impeded control of precision grasping. This occurred particularly in the lifting phase, during which somatosensory signals were important for controlling the grip force^14^. Thus, the present result regarding activation in the PMd during soft incisal biting may indicate that force control according to somatosensory information is important in dexterous incisal biting.

The superior frontal gyrus (BA9) is thought to be associated with voluntary, not automatic, motor control^35^. In a previous fMRI study using the precision-grip task, activation of BA9 was higher when the target force levels were varied compared with when they were constant^36^. Hence, our observation of activation in the superior frontal gyrus (BA9) during soft incisal biting may indicate that intentional control of bite force was required rather than automatic biting.

The caudate is part of the basal ganglia, and the basal ganglia are part of a crucial circuit involved in force production and force selection^37^. In diseases that affect the basal ganglia (e.g. Parkinson’s disease), the ability to regulate force is impaired^38^. Previous fMRI studies have shown that caudate is activated when participants imagine and adjust their precision-grip force^15,39^. Therefore, the present result regarding activation in the caudate indicates that the intrinsic force control system may be engaged during the soft incisal biting task.

The present results showed that cortical activation in the CMAr, superior frontal gyrus (BA 6 and 9), and caudate during incisal biting linearly decreased as EMG activity increased. However, when we checked the beta values for the contrast of incisal biting task at the anatomical ROIs, defined in the bilateral anterior cingulate gyrus (right; 11/15 participants, left; 13/15 participants), bilateral superior frontal gyrus (right; 9/15 participants, left; 9/15 participants), and the bilateral caudate nucleus (right; 6/15 participants, left; 7/15 participants), the values were negative even in the soft force condition, in which the strongest activation was observed. Thus, whether these regions play roles in fine motor control function, or alternatively, if the function of these regions was merely reduced, is not clear.

A neural trajectory study in macaque monkeys showed that the CMAr projects to the oro-facial motor area in the SMA^40^ and to the primary motor cortex^41^, which both project to the brain stem and are involved in rhythmical jaw movement (rhythmical chewing). In the owl monkey, microstimulation in the rostral part of the BA6 (PMd) evoked face and neck movement^42^. Furthermore, a human fMRI study found BA6 activation during gum chewing^43^. Regarding the caudate, a previous fMRI study reported caudate activation during rhythmical gum chewing^44^. Therefore, these three regions appear to be involved in rhythmical chewing. Rhythmical chewing is generally performed in association with molar biting. Indeed, we found positive activation in the CMAr, superior frontal gyrus, and caudate during molar biting, indicating that rhythmical chewing was performed. Hence, we might have found negative activation in these three regions during incisal biting because rhythmical chewing is not generally used in conjunction with daily incisal biting, and the function of rhythmical chewing is inhibited. However, some participants exhibited positive BOLD signals during soft incisal biting, possibly because these regions manipulated fine motor control in the soft incisal biting condition.

As a potential limitation of the present study, the biting task we employed did not mimic a natural chewing movement because we specified the timing of biting as jaw movement at 0.33 Hz. Different results might be obtained using a task that is more similar to natural mastication, in which timing is not specified. Another possible limitation is that we did not perform experiments with power and precision grip, although we have interpreted the present results in comparison to handgrip. However, we believe it is reasonable to discuss the results of the present biting task in comparison with a grasping task, because they exhibit similar brain activation patterns corresponding to the force levels, which covaried with exerted force, in accord with the findings of several previous studies^12–15^.

Regarding the implications of the present study, observing the cortical motor control systems may provide a novel strategy for evaluating the peripheral function of incisors and molars while actually operating in terms of brain function. In addition, our present results may help to clarify how occlusal hypofunction or dysfunction affects cortical motor control systems. Furthermore, the present results may help to elucidate the mechanisms underlying the rehabilitation of peripheral masticatory function via dental treatment, such as prosthetic and orthodontic treatment, and its effects on the cortical motor control systems.

In conclusion, our results indicate that cortical activation, particularly in the M1S1 and cerebellum, is linearly and positively correlated with EMG activity during molar biting, whereas cortical activation, particularly in the CMAr, superior frontal gyrus, and caudate nucleus, is linearly and negatively correlated with EMG during incisal biting. These results suggest that, considering the analogy of hand grasping behaviour, molars engage in powerful chewing: brain activity in several brain areas related to motor function was increased with increasing bite force levels, similar to a power grip. In contrast, incisors participate in dexterous biting: brain activity in the other brain areas related to fine motor control was reduced with increasing bite force levels, similar to precision grip. Consequently, we suggest that motor control system of masticatory movement may be different between incisal and molar biting.

## Methods

### Participants

Fifteen healthy volunteers (10 males and 5 females, mean age = 33.8 years, range 26–63 years) participated in this study. Fourteen participants were right handed and one was left handed according to the Edinburgh handedness inventory^45^. All participants had normal occlusion with absence of obvious defects of the occlusal surface and any missing teeth. None of the participants had a history of neurological or psychiatric illness. The protocol was approved by the ethical committee of the Tokyo Medical and Dental University (approval D2013-009). We confirmed that all methods were performed in accordance with the relevant guidelines and regulations. The participants were informed in detail about the nature of the experiment, and gave their written informed consent for the study.

### Experimental apparatus and task

For each participant, we constructed two mandibular occlusal splints using individual articulators. Dental casts of the upper and lower jaw of each participant were mounted using occlusal records such that there was a 5.0 mm distance between the upper and lower incisors. A silicon patty was positioned on the occlusal surface of each splint such that participants were force to bite only with their incisors (central and lateral incisors on both sides) or molars (second premolars, first molars, and second molars on both sides), respectively.

Participants wore either of the two splints, and bit them at a constant frequency of about 0.33 Hz in synchronisation with the timing of visual cues (NBS Presentation, Neurobehavioral Systems, Inc., Berkeley, CA), such that the circle presented on the screen flashed at the same frequency. To measure different biting force intensities, the participants were asked to bite using three different force levels (soft, medium, and hard). Specifically, they were asked to exert a force corresponding with 20%, 50% and 80% of their maximum bite force in the soft, medium, and hard conditions, respectively^46^. They practiced the task before the scanning procedure to ensure accurate execution. In the practice period, they performed the incisal and molar biting tasks outside of the MRI scanner in response to the visual cues used in the experimental condition. During the practice periods, we recorded EMG from the masseter and temporal muscles using a K7 evaluation system (Myotronics-Noromed Inc., Seattle, WA). This provided the participants with real time visual feedback about their actual EMG activity. After the practice period, we confirmed that the participants had mastered the task and were able to produce three easily distinguishable bite force levels (soft, medium, and hard). During the experimental period, we did not present visual feedback regarding the exerted force.

### Data acquisition

The participants completed two scanning runs, one involving incisal biting and the other involving molar biting. Each run consisted of ten rest-blocks and nine chew-blocks presented in an alternating pattern, and each force-level condition was performed three times. Each block was 21 s in duration. One chew-block consisted of seven biting cycles, and one biting cycle consisted of 1.95 s of isometric biting and a 1.23 s rest period. As mentioned above, visual cues were presented throughout the scanning run, even in rest-blocks (grey colour cues). Three bite force levels (soft, medium, and hard) were indicated by the different colour cues (red, yellow, and blue, respectively) in each biting-block. The two scanning runs were performed on the same day and the biting type order was randomized.

During each fMRI scanning run, as in our previous EMG-fMRI experiment system^47,48^, EMG was simultaneously recorded from the bilateral masseter and temporal muscles using BrainAmp ExG MR (Brain Products, Gilching, Germany). The amplifier and MRI scanner were synchronised using SyncBox (Brain Products, Gilching, Germany). Surface electrodes were placed over the bilateral masseter and temporal muscles with an inter-electrode distance of ~2 cm. EMG signals were fed into a battery-driven amplifier placed on the scanner bed. EMG data were sampled at a digitisation rate of 5 kHz with an amplitude resolution of 0.5 μV/bit and a dynamic range of 16 mV.

During the fMRI recordings, we acquired 135 volumes in each scanning run using T2* weighted gradient echo-planar imaging (EPI) sequences with a 3.0 Tesla scanner (Trio 3T MRI system, Siemens Healthineers, Erlangen, Germany). Each volume consisted of 44 interleaved slices that were 3.0 mm thick without a gap. The time interval between two successive acquisitions of the same slice was 3000 ms, with a flip angle (FA) of 90°and echo time of 30 ms. The field of view (FOV) was 192 mm and the in-plane matrix size was 448 × 448 pixels. For anatomical reference, T1-weighted magnetization-prepared rapid-gradient echo (MPRAGE) images (TR = 2000 ms, TE = 4.38 ms, FA = 9°, FOV = 192 mm, matrix size = 176 × 192 pixels, slice thickness = 1.0 mm) were obtained for each participant using identical location parameters.

### EMG data analysis

**Figure 5:**
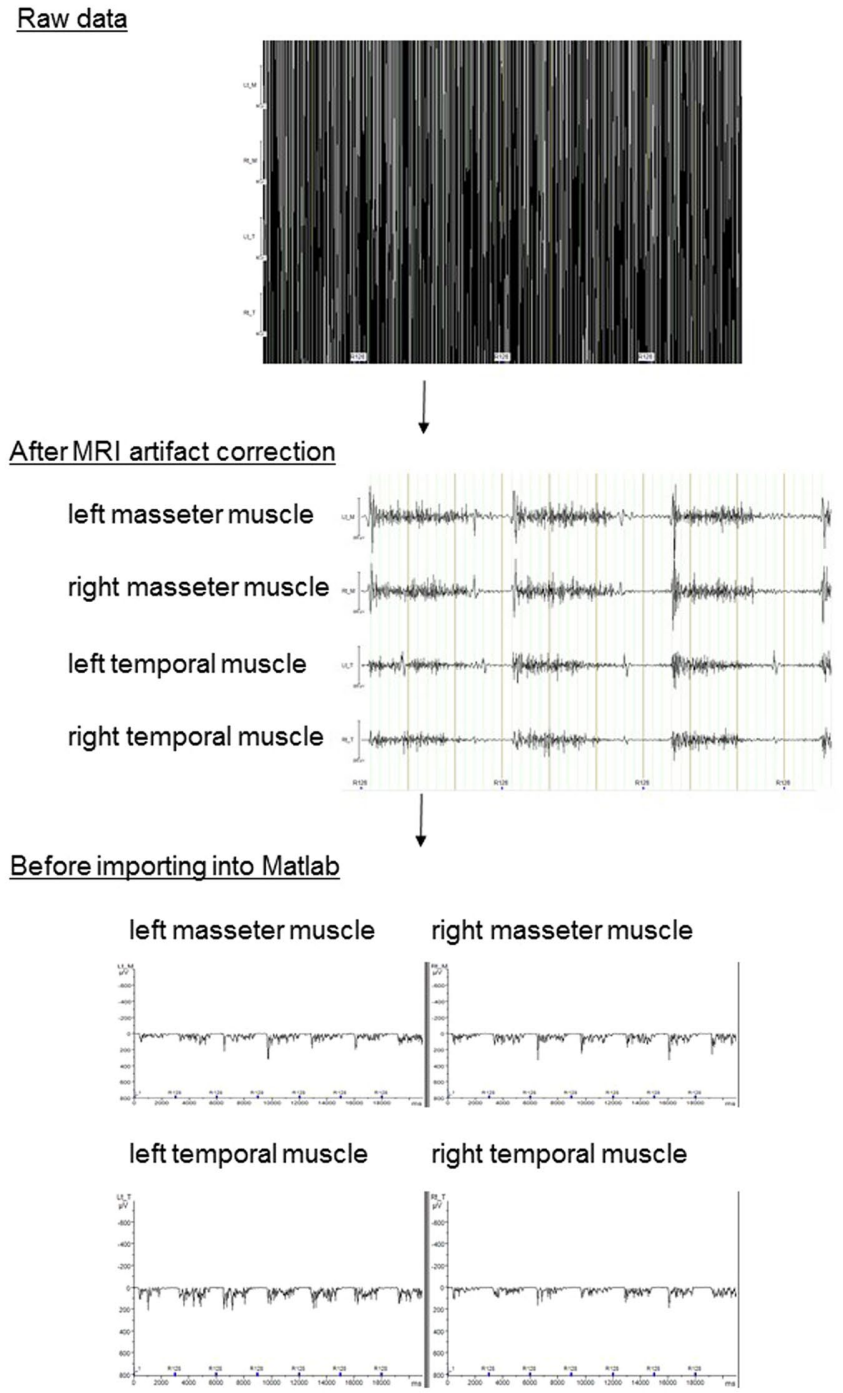
A representative data describing the process of EMG data analysis. The raw data (top) were corrected using a high-pass filter and by MRI artefact correction. After artefact correction (middle), the EMG signals collected in each block were rectified and imported into Matlab for further analysis (bottom).

We used Brain Vision Analyzer software (version 2.1; Brain Products, Gilching, Germany) to correct the data for scanner artefacts. First, we applied a high-pass 10 Hz filter to remove possible movement artefacts. MRI artefact correction was performed using a 200 Hz low-pass filter^47^. After artefact correction, the EMG signals collected in each block were processed via full-wave rectification. Finally, the data were imported into Matlab (Mathworks, Sherborn, MA) for further analysis. Figure 5 shows representative data describing the process of EMG data analysis. We then calculated the average integrated value^48^ per one bite, i.e. the integrated value per 1 block divided by 7.

For statistical analysis, we used a 3 (bite force level: soft/medium/hard) × 2 (biting position: incisor/molar) two-way repeated ANOVA and conducted post hoc analyses using a paired t-test accompanied by the Bonferroni-Holm correction. If we detected an interaction between two variables, we conducted a simple main effects test for each variable, adjusted via the Bonferroni multiple comparisons test. All statistical procedures were performed using commercial statistical software (SPSS Release, Chicago, Illinois, USA). A threshold of P < 0.05 was considered to indicate statistical significance for two-way repeated ANOVA and simple main effects test, and adjusted P-values were compared with 0.05 using a paired t-test with a Bonferroni-Holm correction.

### fMRI data analysis

To verify the hypothesis in the current study, we conducted ROI analysis for the hypothesised brain regions and compared the change in brain activation corresponding to the bite force levels between two biting tasks. We used SPM12 implemented in Matlab to obtain the beta values that represented the intensity of correlation between brain activation and EMG activity.

#### Preprocessing

The first two volumes of functional images in each scanning run were discarded due to unsteady magnetization. The remaining 133 volumes per run (266 volumes per participant) were used for analysis. We corrected the time lag of each scan in a volume using a slice timing correction program with the middle slice as a reference slice. Head motion was corrected using the SPM12 realignment program. According to the realignment parameter, we confirmed that inter-scan changes of head position were less than 1 mm in all participants. Thus, we considered that head motion within every scan was less than 1 mm. Following realignment, T1-weighted MRI scans were coregistered to the functional mean images created in the realignment program. We estimated the parameters for affine and nonlinear transformation into a template of T1-weighted images already fitted to the tissue probably map (TPM) template with the coregistered anatomical T1-weighted MRI scans. The parameters were applied to the coregistered functional images. Spatial smoothing was applied at 8 mm × 8 mm × 8 mm full width at half maximum.

A general linear model^49^ was used to remove the effects of confounding factors. In this procedure, we applied the realignment parameters describing the head position during the scans to the design matrix as multiple regressors. Moreover, we used an epoch-related parametric study design, which included the actual EMG activities of the masseter and temporal muscles as regressors (“parametric modulation” in SPM^50^). This allowed us to distinguish between regions that were active during biting, irrespective of EMG activity, and regions for which activity covaried with the EMG activity. We summed the EMG activities of the two masseter muscles (left and right) and two temporal muscles (left and right), and Z-score normalized the data points within participants to use them as parameters of EMG activity in the masseter and temporal muscles, respectively.

#### ROI analysis

Template masks were drawn a priori from the anatomical volume of interest (VOI) based on the AAL atlas, and were therefore independent of the voxel-wise results^51^. We examined the bilateral precentral gyrus and posterior cerebellum, which were reported to exhibit activation that increased with increasing force levels in previous studies using power-grip tasks^16–18^. Moreover, we examined bilateral anterior cingulate gyrus (including the CMAr), which was reported to exhibit activation that increased with reducing force levels in a previous study using precision-grip task^19^. In addition, we focused on bilateral superior frontal gyrus, caudate, and putamen, which have been reported to be associated with fine motor control in previous handgrip studies using precision-grip tasks^13–15^.

We obtained the beta values for INC-co and MOL-co using the SPM toolbox MarsBar 0.44 (MRC Cognition and Brain Sciences Unit, Cambridge, UK) for ROI analysis in all participants. The averaged beta values over all voxels of the ROI were calculated for each ROI, and used for further statistical analysis. We then compared beta values of INC-co and MOL-co for each ROIs using a paired t-test (significance at P < 0.05).

#### Differences in brain activity covaried with EMG activity between incisal and molar biting

To check whether the anatomical ROIs we selected were in reasonable regions, we compared activation showing positive or negative linear correlations with EMG activity during incisal and molar biting for illustration purposes. Specifically, we contrasted MOL-co vs. INC-co and vice versa.

## Data Availability

The data supporting the findings of this study are available from the corresponding author, MJJ, upon request.
